# Hyperleukocytosis in a neonate: A diagnostic dilemma

**DOI:** 10.4103/0971-5851.73596

**Published:** 2010

**Authors:** Sushanth, K. Shreedhara Avabratha, Kiran Joseph Tauro, G. K. Shwethadri

**Affiliations:** *Department of Pediatrics, Father Muller Medical College, Mangalore, Karnataka, India*; 1 *Department of Pathology, Father Muller Medical College, Mangalore, Karnataka, India*

**Keywords:** *Congenital leukemia*, *hyperleukocytosis*, *leukemoid reaction*, *transient myeloproliferative disorder*

## Abstract

A preterm baby presented with lethargy and tachypnea. Blood counts revealed hyperleukocytosis. Peripheral smear and bone marrow examination were not suggestive of leukemia. The baby was treated for sepsis. The baby recovered and WBC counts gradually reduced. Hyperleukocytosis was presumed to be a part of leukemoid reaction secondary to sepsis. The diagnostic possibilities with a review of literature are also presented.

## INTRODUCTION

Leukocytosis refers to an increase in the total number of white blood cells due to various physiologic, infectious, inflammatory or malignant processes. Hyperleukocytosis, defined as a total WBC count of >100,000/mm^3^, usually occurs in leukemia and other myeloproliferative disorders. Physiologic leukocytosis is well recognized in the neonate but counts rarely exceed 30,000/mm^3^.[1] We report a case of hyperleukocytosis in a preterm neonate and discuss the diagnostic possibilities with a review of literature.

## CASE REPORT

A preterm male baby (30–32 weeks of gestation) born to a primigravida mother by normal vaginal delivery was referred for preterm care on Day 1. The mother had regular antenatal checkups and had not received antenatal steroids. There was no ABO or Rh incompatibility. On examination, the baby weighed 1.66 kg and was lethargic. He had tachypnea with intercostal retractions. Liver was palpable 2 cm below the right costal margin.

**Figure 1:**
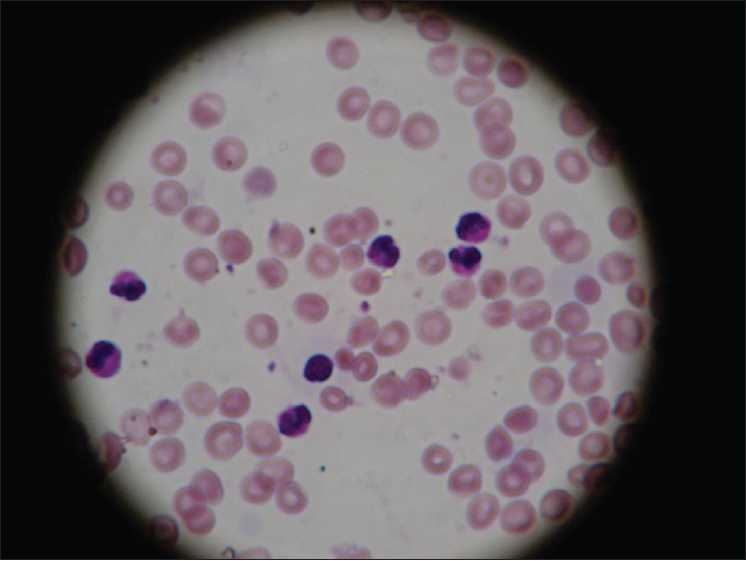
Peripheral smear showing neutrophils and band cells (×400)

**Figure 2:**
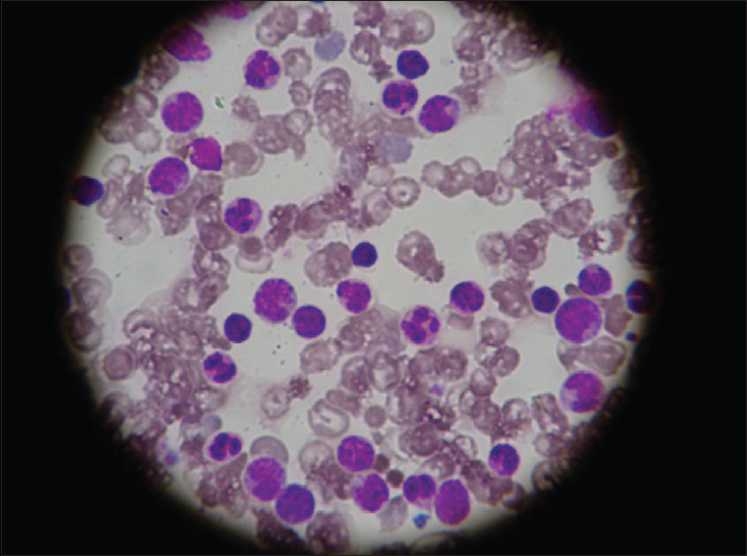
Bone marrow showing predominantly mature neutrophils (×400)

Investigations revealed the following: Hb 12.3 g%, WBC count 98,200/mm^3^, peripheral smear 15% promyelocytes, 10% myelocytes, 18% band forms, 35% neutrophils 2% eosinophils, 5% lymphocytes and 20 nRBC/100 WBC [Figure 1]. His platelet count was 246,000/mm^3^and he had an elevated C-reactive protein (CRP) level at 56.8 mg/L. The baby was started on supportive care and IV antibiotics. WBC counts repeated at 48 hours of life showed an increasing trend at 145,900/mm^3^with predominant neutrophils. Peripheral smear failed to demonstrate any abnormal cells or blasts. Serum lactate dehydrogenase (LDH) was elevated (1556 U/L). Bone marrow aspiration done on the fourth day was normal [Figure 2]. Karyotyping was done which showed a normal 46 XY pattern. Blood culture grew methicillin resistant *Staphyloccus aureus* (MRSA) and antibiotics were changed based on the sensitivity pattern.

The patient was closely monitored for complications of hyperleukocytosis like intracranial hemorrhage, respiratory failure, hyperuricemia and renal failure. The WBC counts of the patient were closely monitored which showed a gradual reduction and was 10,000/mm^3^on the 15th day of life. The patient responded well to supportive care and was discharged after 23 days of hospital stay. On follow-up, he has been asymptomatic with adequate weight gain and has WBC counts within the normal range.

## DISCUSSION

Leukocytosis is a commonly encountered finding in a neonate. The normal leukocyte count in a neonate ranges from 9000 to 30,000/mm^3^.[1] This physiologic leukocytosis appears to be due to a surge in cytokine secretion (Granulocyte Colony Stimulating Factor and Granulocyte Macrophage Colony Stimulating Factor) in the immediate postpartum period.[2]

Leukemoid reaction is a moderate, advanced, or sometimes extreme degree of leukocytosis, which is similar to that occurring in leukemia but is due to some other cause. Conventionally, a leukocytosis exceeding 50,000 WBC/mm^3^with a significant increase in early neutrophil precursors is referred to as leukemoid reaction. There is a mix of early mature neutrophil precursors, in contrast to the immature forms typically seen in leukemia. Incidence of leukemoid reaction in the N ICU varies from 1.3 to 15%.[3] The most common causes include antenatal administration of betamethasone, infection and transient leukemoid reactions of Down syndrome.[3] A retrospective review of a series of preterm infants has demonstrated leukemoid reactions occurring in up to 15% of preterm neonates in the absence of an identifiable cause.[2] However, leukemoid reaction severe enough to cause hyperleukocytosis is very rarely encountered and reported in literature.

Hyperleukocytosis is defined as WBC counts>100,000/mm^3^and most cases encountered are due to either congenital leukemia or a transient myeloproliferative disorder occurring in association with Down syndrome, both of which are relatively rare entities.

Congenital leukemia is a term used to describe leukemia diagnosed at birth or in the first month of life. It is a rare entity with a majority of cases being of myeloid origin, commonly either monoblastic or myelomonocytic.[3,4] The clinical presentation is defined by leukocytosis, hepatosplenomegaly, central nervous system involvement and skin manifestations (Blueberry muffin like rash). However, the diagnosis is established by the demonstration of blasts in the peripheral blood and the bone marrow. The absence of an orderly morphologic progression between the blasts and the mature cells (so called “Leukemic Hiatus”), along with very highly elevated LDH levels can help to differentiate leukemia from leukemoid reactions.[5]

Transient myeloproliferative disease (TMPD), also known as transient abnormal myelopoiesisis, is a form of transient leukemia encountered in around 10% of cases of Down syndrome.[6] The clinical presentation can vary from asymptomatic neonates with incidentally discovered leukocytosis (25%) to cases presenting with features of leukemia. Spontaneous resolution is noted in up to two-third of the cases by 12 weeks of age. Although they were initially thought to occur exclusively with Down syndrome, a few cases of them occurring in infants with a normal karyotype have been described.[7] The peripheral smear and bone marrow examination reveals a large population of blasts with a leukemic hiatus. Although the presence of thrombocytosis in TMPD can help differentiate it from leukemia, spontaneous resolution of the hematological features clinches the diagnosis. The recent detection of GATA-1 mutation in cases of TMPD can prove to be a useful diagnostic tool in the future.[8] This condition is now considered as a preleukemic state with a risk of development of leukemia within the first 4 years of life.[9] Management consists of close observation and treatment of complications like hyperleukocytosis. A subset of symptomatic patients may benefit from low dose cytarabine to achieve remission.

Hyperleukocytosis with high LDH level in our patient prompted us to consider the possibility of congenital leukemia and TMPD. However, the absence of blasts in both the peripheral smear and the bone marrow and the normal karyotype helped us to exclude them. The hyperleukocytosis seen in our case was probably due to leukemoid reaction secondary to staphylococcal sepsis, which is an unusual presentation. Further, the WBC counts promptly normalized in response to appropriate antibiotics. However, we would like to emphasize that a thorough workup and regular follow-up to rule out the possibility of leukemia is indicated in all cases of hyperleukocytosis.
